# A Task-Driven Feedback Imager with Uncertainty Driven Hybrid Control †

**DOI:** 10.3390/s21082610

**Published:** 2021-04-08

**Authors:** Burhan A. Mudassar, Priyabrata Saha, Marilyn Wolf, Saibal Mukhopadhyay

**Affiliations:** 1 School of ECE, Georgia Institute of Technology, Atlanta, GA 30332, USA; burhan.mudassar@gatech.edu (B.A.M.); priyabratasaha@gatech.edu (P.S.); 2 Deparment of CSE, University of Nebraska-Lincoln, Lincoln, NE 68588, USA; mwolf@unl.edu

**Keywords:** smart camera active sensors, feedback control, deep neural network (DNN), object detection, action detection, uncertainty estimation

## Abstract

Deep Neural Network (DNN) systems tend to produce overconfident or uncalibrated outputs. This poses problems for active sensor systems that have a DNN module as the main feedback controller. In this paper, we study a closed-loop feedback smart camera from the lens of uncertainty estimation. The uncertainty of the task output is used to characterize and facilitate the feedback operation. The DNN uncertainty in the feedback system is estimated and characterized using both sampling and non-sampling based methods. In addition, we propose a closed-loop control that incorporates uncertainty information when providing feedback. We show two modes of control, one that prioritizes false positives and one that prioritizes false negatives, and a hybrid approach combining the two. We apply the uncertainty-driven control to the tasks of object detection, object tracking, and action detection. The hybrid system improves object detection and tracking accuracy on the CAMEL dataset by 1.1% each respectively. For the action detection task, the hybrid approach improves accuracy by 1.4%.

## 1. Introduction

A critical component of data acquisition in active sensors [1,2,3,4] is the controller that modules the output of the sensor. As Artificial Intelligence (AI) and Machine Learning (ML) components become ubiquitous, they are increasingly being used as part of the controller [2,3,4]. These design decisions have ramifications for the downstream systems that consume and act on this sensor data. Thus, it is important to establish measures of reliability and trust, especially when a DNN becomes a part of the controller [5,6,7].

Modern DNN-based task architectures are by nature black-box systems and do not lend themselves to introspection. Moreover, it is shown in many studies that the DNNs tend to generate uncalibrated overconfident detections [8]. Nevertheless, a body of research exists in quantifying the uncertainty of the DNN output through a mix of sampling and non-sampling based approaches [9,10,11]. However, there does not exist any studies on the uncertainty behavior of DNN controllers in an active sensor system.

In this paper, we pick a closed-loop imager with a task DNN embedded in the control loop [4] and study it from the lens of uncertainty estimation. We examine a number of tasks including object detection, object tracking, and action detection. The task DNN outputs Regions of Interest (ROIs) which are then used to guide the sampling characteristics of the sensor array. The sampling characteristics include the spatial resolution of the pixel array, the temporal sampling rate. In addition to that, we also examine a multi-modality imager [3]. The imager uses the confidence of the detections to decide which of these will be used for feedback.

**Figure 1:**
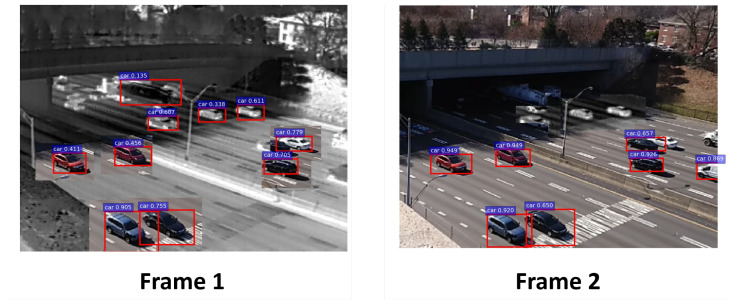
Problems with confidence-driven control in a multi-modal sensor. The truck on the top region of the frame gets rejected due to a low confidence (0.135). The detection, however, has a high level of uncertainty. The uncertainty information can be exploited to keep this region in IR.

The problem with this kind of confidence-driven control is that relying just on the score of the detection can lead to both false negatives (FN) and false positives (FP). A visual example in Figure 1 shows a false negative with a low confidence score in the IR domain. Thus, it is not kept as ROI for the next frame, and the area in question switches to RGB. However, the uncertainty of that detection is higher in relation to the other ROIs. The uncertainty measure gives an indication that the network may have made a mistake and that ROI needs to be kept. This observation motivates us to propose a hybrid control system that uses both the score of the detection as well as its uncertainty to decide the control action.

This paper extends our work on uncertainty characterization and the hybrid feedback loop [12] presented earlier. We present additional settings of the hybrid control scheme that work well on false negatives in addition to false positives. We also scale the concepts to harder tasks. Specifically, we apply it to tasks with a well-defined temporal dependence e.g., action detection. We show a procedure for separating out the contribution of spatial and temporal features to the total uncertainty. In addition, we show that, in a practical setting, estimating just the temporal uncertainty leads to a good performance when considering system constraints in addition to the accuracy. Overall, this paper makes the following contributions:

- We characterize the uncertainty of a closed-loop imager with an embedded task DNN in the control loop. We show that uncertainty is closely linked to accuracy of the system, and, in the absence of ground-truth information, it can be used as a proxy measure of success.
- We study the effect of input perturbation, such as additive Gaussian noise, on the uncertainty. In addition, we also characterize the uncertainty for different input modalities such as RGB, IR.
- We propose a feedback system that uses uncertainty in addition to the confidence of the detections. The hybrid system removes over-confident detections and allows the imager to focus on areas of the image where it is uncertain about its performance.
- We propose a methodology for establishing causality of the uncertainty on spatial or temporal features for the task of action detection.

**Figure 2:**
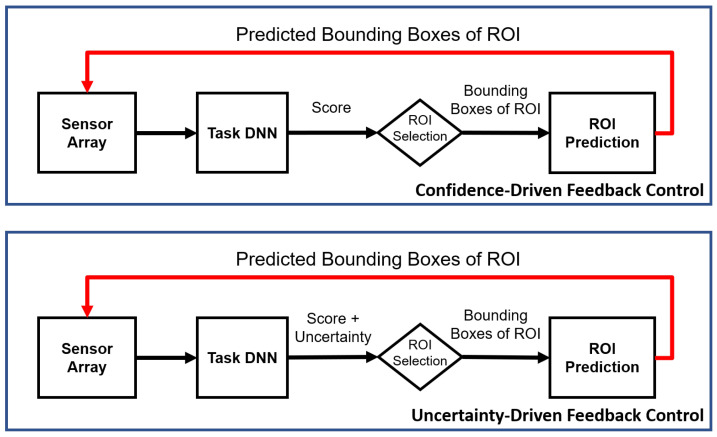
Proposed feedback system with uncertainty as an additional criteria for applying feedback.

The new uncertainty-driven control (Figure 2) improves performance for false negatives on the CAMEL dataset [13] for the tasks of object detection and tracking. The CAMEL dataset contains RGB-IR videos with pedestrians and cars annotated. The uncertainty control improves AP over the mixed-modality baseline by 1.1%. Similarly, for the action detection task, the uncertainty-driven control improves frame mAP by 1.1% over the confidence-driven control on the UCF-Sports Dataset [14]. Finally, by separating the spatial and temporal uncertainty, we can achieve the same accuracy metrics at a fraction of the original cost of multiple Monte Carlo (MC) trials.

The rest of this paper is organized as follows: Section 2 presents prior work in literature. Section 3 presents a review of uncertainty estimation techniques in DNN and the feedback imager. Section 4 presents the prior confidence-driven feedback imager. Section 5 and Section 6 present the confidence and uncertainty-driven feedback and the separated spatial and temporal uncertainty. Section 7 and Section 8 present the experimental results.

## 2. Related Work

Feedback is an essential component of any imager. In commercial images, heuristic-driven feedback loops are present such as Auto-White Balance, Auto-Exposure, etc. The purpose of these control loops is to shift the captured image statistics towards the statistics of natural images and to generate a high-quality image for human perception. In this work, our focus is to tune the image to improve the accuracy of an end-user task such as pedestrian detection. There exists prior work on task-driven feedback. They are discussed below.

### 2.1. Task-Driven Feedback

Early works in task-driven feedback focus on reducing the bandwidth required to transmit the pixels from the sensor by selectively reading out some pixels. For example, Chalimbaud et al. proposed selective readouts of the frame using output from an object tracker [1]. Other works do compression of the pixels based on the task output. For example, Wells et al. perform compression of sensor pixels in the DCT domain with direct feedback from a spatial and temporal engine. The spatial and temporal engines use DCT coefficient importance and motion estimation-compensation outputs, respectively, to drive the control [15]. Going further, Wells et al. sample foreground pixels at full resolution and adaptively decrease the resolution of background pixels using a tree configuration. The sampling is driven by a segmentation and object tracking algorithm [16]. PISP performs adaptive video encoding driven by motion estimation and motion compensation algorithms to reduce the impact of soft errors in video compression [17]. Ko et al. use a multi-QF JPEG encoder to encode motion ROIs at a higher QF while meeting bandwidth constraints [18].

While the above works focus on task-driven compression, there also exists prior work which performs control directly at the sensor level. This control either changes the spatial resolution, temporal resolution, pixel depth, or the spectral modality of the pixels of interest. For example, Saha et al. propose an early fusion of IR and Visual sensor modalities using the output of an object detection task [3]. Saha et al. propose a Reinforcement Learning (RL) based controller for multispectral fusion [19]. Mudassar et al. propose spatio-temporal resolution control of individual pixels in a digital pixel sensor (DPS) using the output of object detection and action detection tasks [4]. Mukherjee et al. propose a cross-layer control scheme for tuning the pixel depth of ROI and non-ROI pixels [20]. What these works do not address, however, is the uncertainty in the task output itself. In this work, we apply uncertainty estimation techniques on the task and use those to filter the ROIs provided to the controller.

### 2.2. Uncertainty Measurement in DNN

DNN models generate point estimates in their default configurations but do not produce uncertainty for each input sample. There exists prior work that focuses on modifying the DNN to produce uncertainty estimates. Broadly, existing techniques can be classified as sampling-based [9,10] or non-sampling based [11]. Sampling-based techniques perform inference multiple times on random permutations of the network. MC Dropout [10] randomly turns off neurons in the network. Bayes By Backprop [9] samples from the learned weight distributions. In contrast, non-sampling based techniques try to learn the variance of the predictive variables during training by attenuating the loss with the learned variance [11]. We apply both sampling-based and non-sampling based approaches in our work to measure the uncertainty of the task DNN.

### 2.3. Uncertainty Estimation for Detection Tasks

Uncertainty techniques for detection tasks [21,22] also involve a combination of sampling-based methods [23,24,25] and non-sampling-based methods [26,27,28]. Miller et al. evaluate the performance of object detectors in open-set conditions. They show that a sampling-based dropout object detector is better at rejecting false positives and lowering the overall uncertainty [23]. They also evaluate merging strategies for aggregating detection outputs across multiple samplings, e.g., based on spatial affinity, based on same winning class label, etc. [21]. Hall et al. propose a new metric for measuring detection uncertainty called Probabilistic Detection Quality (PDQ) [22]. Non-sampling based techniques add a learned variable of uncertainty during the training process. Effectively, the output class and bounding box variables have a counterpart uncertainty variable that is learned and is dependent on the input. He et al. learn variance of the bounding boxes and use variance voting to reduce importance of high variance boxes [26]. Wirges et al. apply loss attenuation to learn variance of 3D bounding boxes in 3D object detection [27]. Corbiere et al. use true class probability instead of maximum class probability during the training process [28].

## 3. Background

**Figure 3:**
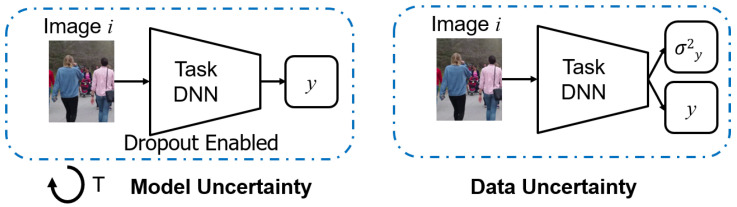
Method for calculating Model and Data Uncertainty in DNN. In model uncertainty, inference is run for *T* trials with dropout enabled. The sample variance is then computed. In data uncertainty, the variance is computed as a function of the input and the weights of the network. Reprinted with permission from ref. [12]. Coprright 2020 IEEE.

The uncertainty estimates for the task DNN are collected using both a sampling [10] and a non-sampling method [11] referred to as model and data uncertainty, respectively (Figure 3). The model uncertainty or epistemic uncertainty model the uncertainty in parameters of the model. It can be reduced by increasing the training data or adding more knowledge. Hence, it is also known as knowledge uncertainty. The data uncertainty or aleatoric is due to the inherent noise or irregularities in the signal which cannot be removed by adding more data. An example is an occlusion which can hinder detecting an object or at least make it harder to ascertain its actual size.

### 3.1. Predictive Variables

In the detection task (object/action), two types of output variables are predicted, i.e., labels and bounding boxes. Both are treated separately when determining their uncertainty. They will be referred to as label uncertainty and location uncertainty. Note that they are separate from model and data uncertainty. We will be calculating model and data uncertainty for both labels and bounding boxes.

### 3.2. Model Uncertainty

In the sampling method, dropout layers are added within the network architecture. The dropout randomly turns off neurons during the forward pass of the network. For a selected input, inference is run multiple times and the predictive variance of the detections is generated through Monte Carlo (MC) averaging. The object detector outputs bounding boxes and class scores. The class uncertainty $\sigma_{cls}$ is computed using the entropy of the mean softmax vector $\mu_{cls}$ (Equation (1)) while the box uncertainty is modeled as a Gaussian RV. The bounding box uncertainty is computed independently for the four bounding box coordinates x,y,w,h using Equation (2), which represents the variance. *T* denotes the number of MC trials. *C* is the number of output classes. *f* represents the DNN, while μ is the mean of the the output variable over *T* trials:

(1) $$\sigma_{cls}=-\sum_{i=1}^{C}\mu_{cls}\left[i\right]*log\left(\mu_{cls}\left[i\right]\right)$$

(2) $$\sigma_{out}=\frac{1}{T}\sum_{i=1}^{T}f^{\left(i\right)}{\left(in\right)}^{T}f^{\left(i\right)}\left(in\right)-\mu_{out}^{T}\mu_{out}$$

### 3.3. Data Uncertainty

Aleatoric uncertainty is estimated by directly learning to predict variance parameters for each output variable. The cost function is also modified. For bounding box regression, the loss is attenuated by the predictive variance σ (Equation (3)). *y* represents the network output while $y_{gt}$ represents the ground truth. A regularization term is also added so that the network does not learn to ignore the training data and predict a high variance of all inputs. The reader is referred to [11] for more details:

(3) $$L_{reg}=\frac{1}{\left(2\sigma^{2}\right)}*||y-y_{gt}\left|\right|+\frac{1}{2}*log\left(\sigma^{2}\right)$$

For classification, the logits vector *x* before the softmax are corrupted by a random vector with variance equal to the predictive variance of each class (Equation (4)). The mean and variance of the output after softmax cannot be analytically computed, so the sample mean and variance is computed by drawing MC samples from the logits and applying the softmax function. The mean of the softmax outputs is computed using Equation (5), and the variance is represented by the entropy of the mean softmax vector:

(4) $$g^{\left(i\right)}\left(x,\sigma_{cls}\right)=f^{\left(i\right)}\left(x\right)+N\left(0,\sigma_{cls}\right)$$

(5) $$\mu_{cls}=\frac{1}{T}\sum_{i=1}^{T}softmax\left(g^{\left(i\right)}\left(x,\sigma_{cls}\right)\right)$$

### 3.4. Tasks and DNN Architectures

For the characterization and uncertainty-driven control, we look at a number of high-level detection-based tasks which include object detection, object tracking, and action detection. The complexity of these tasks requires a DNN-based implementation, so we take off-the-shelf DNN architectures and fine-tune or re-train them for feedback operation and to be able to get uncertainty measures from them.

**Figure 4:**
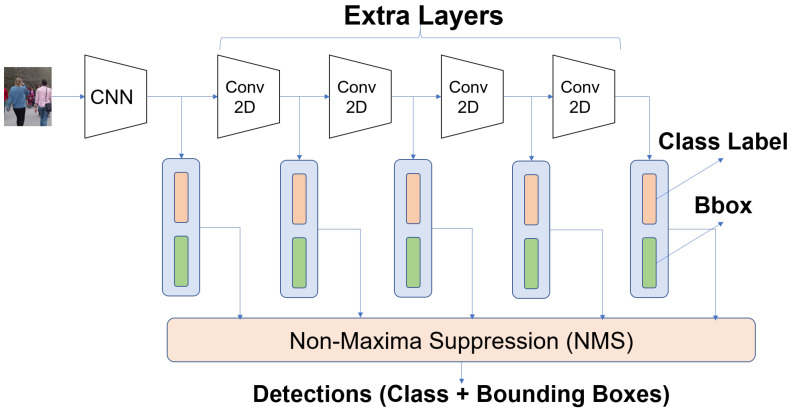
SSD Architecture [29] for Object Detection, which consists of a CNN backbone followed by extra convolutional layers. Classification and Regression Layers follow, and the last step is NMS, which removes redundant detections based on spatial overlap.

Experiments were conducted for the tasks of object detection and object tracking. A SSD-based network architecture was used for both tasks [29] (Figure 4). A Mobilenet-v1 [30] backbone was used for object detection, which is suitable for an embedded implementation. We use the modified version SSD Mobilenet V1 that is more suitable for small object detection [31]. The object tracker is a tracking by detection system, which uses hypotheses from the object detector to perform tracking. We use the SORT tracker [32] for multi-object tracking.

**Figure 5:**
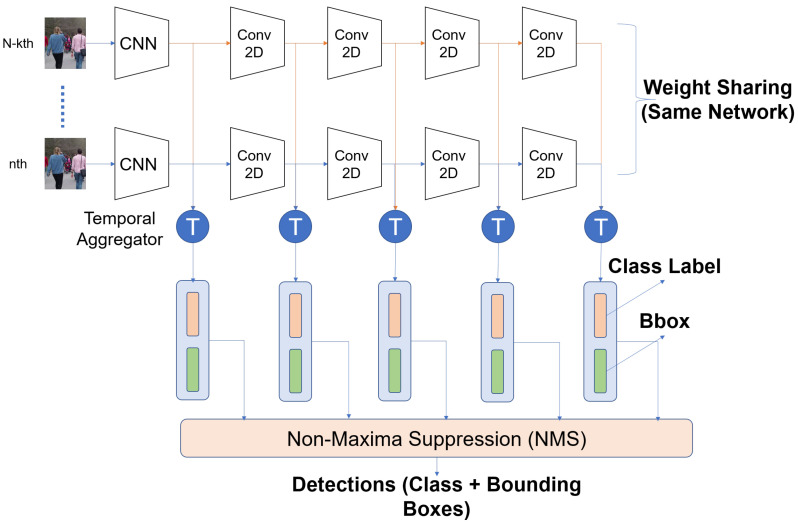
ACT Architecture [33] for action detection. It is similar to SSD, but it takes in multiple frames as input and applies aggregates the features for every time step before doing classification and regression. The network applied to each frame is the same.

For action detection, we evaluated on two architectures ACT (Figure 5). The ACT architecture generates features for each frame by passing them through a CNN. The per-frame high dimensional features are aggregated using a temporal aggregator (1 × 1 conv or LSTM or mean) followed by classification and regression layers. The NMS is performed both per-frame and on a video basis. Both architectures take in multiple frames as input and generate detections. An offline post-processing step links the detections across frames to produce action tubes.

## 4. Confidence-Driven Feedback Control

### 4.1. System Architecture

**Figure 6:**
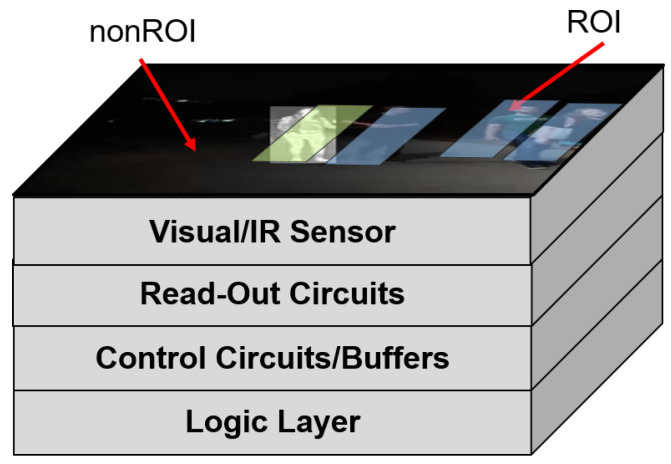
System architecture for the Feedback Imager. A 3D stacked topology allows for the ML control tier to be closely coupled with the sensor tier.

We characterize the uncertainty of a closed-loop imaging system with embedded task-driven feedback [4]. The system consists of a processing-in-memory acceleration tier and a digital pixel sensor array (DPS) in a 3D-stacked topology as shown in Figure 6. A digital pixel sensor array occupies the top 3 tiers. The digital pixel sensor array allows for localized control of each pixel. It is composed of the photodiode tier, a photocurrent to frequency converter (PFC) tier, and a tier containing the counters. Communication between the tiers is realized through high-throughput TSVs and Cu-Cu interconnects [34]. We base the design of our photodiode tier on the broadband array presented by Goosens et al. [35] to realize applications requiring hyperspectral input. The specialized read-out circuits allow for localized control of each pixel. These control functionalities include choosing the modality of each pixel (multi-modality control), choosing which pixels to turn on or off (spatial resolution control), and sampling the pixels at multiple frame rates (temporal resolution control). A logic layer at the bottom of the stack performs real-time task processing to realize the per-pixel control. The confidence-driven feedback control from [4] is used. We call this confidence-based as it solely relies on the confidence of the detections to determine whether they should be used for feedback.

### 4.2. Feedback Control

The detection step is followed by an ROI prediction layer. The ROI prediction module enforces temporal smoothness of detections using a Kalman Filter and predicts locations of objects in the next frame with a linear motion model. The ROIs from the ROI prediction layer drive the feedback control. Two types of control are investigated in this work for uncertainty characterization. The first is spatio-temporal resolution control. In the spatio-temporal resolution control scheme, individual pixels can be turned on or off, or sampled at a lower or faster rate based on a downsampling factor. The second is mixed-modality control in which we create a mixed-modality image where each pixel can be activated in either of the modalities—for example, RGB and IR.

The uncertainty for the baseline system with no feedback and feedback systems with spatial resolution control and mixed-modality control are characterized for the task of object detection and tracking.

#### 4.2.1. Spatial Resolution Control

A downsampling factor $N_{spatial}$ is chosen for spatial resolution control. In this approach, we turn off pixels in local neighborhoods belonging to non-ROI regions. For example, if $N_{spatial}$ is 2, we turn on only one pixel in a 2 × 2 neighborhood. ROI regions are kept at full fidelity. For processing by the DNN, the holes are removed by replicating the chosen pixel.

#### 4.2.2. Temporal Resolution Control

Similar to spatial resolution control, we downsample the temporal resolution of non-ROI regions. The downsampling factor for temporal resolution control is referred to as $N_{temporal}$. This control is used more in the task of action detection as changing the frame interval has a more discernable effect on the detection quality.

#### 4.2.3. Multi-Modality Control

In multi-modality control, a mixed modality image (for example an image with both RGB and IR channel values) is created. However, in one spatial location, it can only be one of either RGB or IR. The control proceeds as follows. For the very first frame of a sequence, an input to object detection network is initialized with any single modality image. An RGB image is used for the first frame, and any detected bounding boxes are considered as RoI. In the next frame, the modality of the RoI is retained to ensure its detectability, whereas the nonRoI modality is switched to IR in search of any missed object. RoIs from the second frame are propagated to the third frame in their respective modalities, whereas nonRoI modality is altered. In this fashion, we keep track of modality for each RoI and ensure they get detected in the next frame while altering nonRoI modality in search of new objects.

In the confidence-driven control, the set of detections $set_{conf}$ is constructed by picking detections that have a score greater than a threshold $th_{conf}$:

(6) $$set_{1}=score\geq th_{conf}$$

## 5. Uncertainty-Driven Feedback Control

The uncertainty-driven feedback control is formulated as follows. In addition to the confidence score of each detection, the uncertainty of the detection (measured using model or data uncertainty) is also used to select it as ROI or non-ROI. As described earlier, we have the label as well as location uncertainty at our disposition. The location uncertainty is useful in the sense that it can correct for mislocalizations of small margins. The label uncertainty is more useful as it allows us to determine whether a misdetection has occurred.

In the uncertainty-driven feedback system, the model/data uncertainty is calculated for each output detection using the methods described earlier. The label uncertainty for each detection is normalized for each frame by the detection having the maximum uncertainty of all detections in that frame. This is done as the entropy fluctuates across frames (although it can be bounded by the entropy of a uniform source). Depending on the values of the uncertainty and the score, we outline the control decisions that need to be taken and are described further.

In the confidence-driven system, we only consider the detections having a high confidence score as correct for feedback. If we incorporate uncertainty into the control, we get four possibilities:

- High Score + Low Uncertainty (True Positive)
- High Score + High Uncertainty (Possibly a False Positive)
- Low Score + High Uncertainty (Possibly a False Negative)
- Low Score + Low Uncertainty (True Negative)

The first possibility corresponds to the score only control as it is likely a true positive. The second possibility is most likely a false positive as the network is overconfident about its prediction. The third possibility can be a false negative as it is missed by the network, but the uncertainty is high. Finally, the fourth possibility is a true negative as both measures are rejecting it. Formally, the hybrid control considers possibility 1 and possibility 3 for providing feedback.

Based on the above hypotheses, we formulate three different types of control that incorporate uncertainty into the feedback pipeline.

### 5.1. Uncertainty-False Positive

In this control, the uncertainty information is used to remove false positives from the decision set (Equation (7)). Detections with an entropy lower than threshold $th_{low}$ and a score greater than $th_{conf}$ are kept:

(7) $$set_{fp}=\left(score\geq th_{conf}\right)\cap \left(entropy\leq th_{low}\right)$$

### 5.2. Uncertainty-False Negative

In this control, the uncertainty information is used to remove false negatives from the decision set (Equation (8)). Detections with an entropy higher than threshold $th_{high}$ and a score lower than $th_{conf}$ are kept:

(8) $$set_{fn}=\left(score\leq th_{conf}\right)\cap \left(entropy\geq th_{high}\right)$$

### 5.3. Uncertainty-Hybrid

In the hybrid control, we merge the two sets to get the decision set for feedback (Equation (9)). The expectation is that this set will balance the removal of false positives and false negatives using the uncertainty information:

(9) $$set_{hybrid}=set_{fp}\cup set_{fn}$$

## 6. Separation of Spatial and Temporal Uncertainty

We introduce formulations for separating the uncertainty contributions of spatial and temporal features. In the current formulation of uncertainty measurement, the uncertainty is measured as a whole and not directly linked to a singular feature or variable. Hence, the causality of the features contributing to the greater uncertainty cannot be established. Particularly for temporal-dependent tasks such as action detection, it cannot be determined whether the spatial features or temporal features are contributing to the increased uncertainty.

If we revisit the ACT architecture, it consists of a CNN followed by temporal aggregation layers and classification/regression layers. The CNN operates independently on each frame to generate per-frame features. The temporal aggregator combines frame features from a set of frames and passes them to a predictor. The detection is done on a whole video in a sliding window fashion.

**Figure 7:**
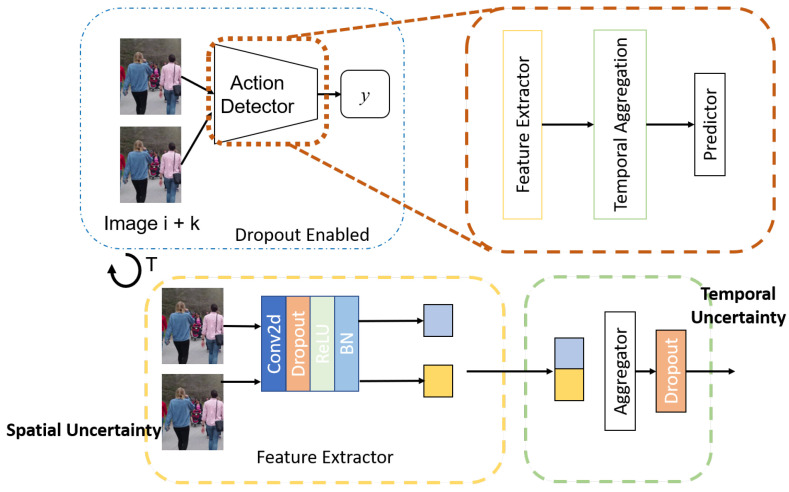
The spatial and temporal uncertainties are separated by changing the insertion points of the dropout layers. In the case of spatial uncertainty, the dropout layers are added in the CNN after every parameter layer. For temporal uncertainty, the dropout layers in the temporal aggregator only.

The uncertainty in the variables is approximated using dropout sampling of the networks. The spatial uncertainty and temporal uncertainty are approximated by changing the insertion points of the dropout layers. The spatial features are generated by the CNN so dropout is added after every parameter layer (Convolution and its variants). Additionally, each frame’s features are generated independently by passing through the CNN, so the notion of temporal behavior is not introduced until the aggregation phase. Thus, to approximate the uncertainty due to the temporal features, the dropout is added after the temporal aggregation (Figure 7).

## 7. Experimental Setup

The SSD Mobilenet v1-S1L0 has a compute complexity of 5.9 GFLOPs. On a Jetson Xavier platform, processing one image takes 26.6 ms for this network [36]. Dropout was added to the output of the backbone with a 0.2 probability of dropping. Evaluation was performed on the CAMEL dataset [13] which contains registered RGB-IR pairs at a resolution of 256 × 336 and 5 classes of objects annotated. The test set contains six sequences with challenging lighting and occlusion conditions. Two datasets were used for benchmarking for action detection. The UCF101-24 [14] dataset contains 928 clips with 24 labeled actions. The MOVE dataset contains 43 clips with 15 labelled actions and a high degree of camera motion.

For each input image, 40 MC trials were performed to get model uncertainty. The threshold for correct detections at the sensor was kept at 0.3, which was the original threshold for the confidence-only controller. The bounding box detections from the trials are clustered using the spatial affinity of the boxes measured using the Intersection over Union (IoU) metric [24]. The class entropy of each detection is normalized by dividing by the maximum entropy among all detections in the same frame.

### Metrics for Evaluation

The diversity of the different tasks requires different measures of success. We provide a preliminary on the important metrics for discussion.

**Average Precision.** The average precision is calculated by measuring the area of the Precision–Recall Curve. The precision and recall values are determined by calculating the number of true positives and false positives. Detections with a high degree of overlap with ground-truth boxes are designated as true positives (TP) while the rest are designated as false positives (FP). Any unmatched ground truth boxes are False Negatives (FN). The detection threshold of the network is swept to get multiple PR pairs. The trapezoidal rule is employed to approximate the area. The AP is measured per-class. For all the classes, the mean AP (mAP) is computed. There are two specializations for the AP based on the task. For action detection frame, mAP and video mAP are calculated. While frame mAP is straightforward, video mAP is slightly different. The video mAP metric uses overlap both over space and time to designate true positive detections. For action detection, video mAP is more valuable as it is important to localize the action both over space and time.

**Multiple Object Tracking Accuracy.** The object tracking task is evaluated using the Multiple Object Tracking Accuracy (MOTA) metric. The MOTA weighs the combined effect of false positives (FP), false negatives (FN), and ID switches (Equation (10)). An ID switch occurs if the running ID for a continuous trajectory changes at some point during the video.

(10) $$\mathrm{MOTA}=1-\frac{\left(\mathrm{FN}\;+\;\mathrm{FP}\;+\;{\mathrm{ID}}_{\mathrm{switch}}\right)}{\mathrm{GT}}$$

## 8. Experimental Results

### 8.1. Characterization

#### 8.1.1. Input Perturbations

**Figure 8:**
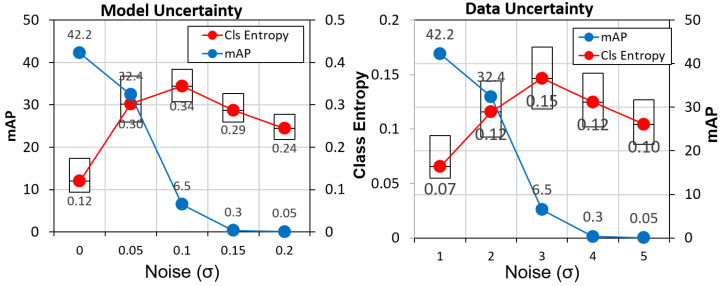
Model and Data Classification Uncertainty estimates for an object detection model on the CAMEL dataset. Reprinted with permission from ref. [12]. Coprright 2020 IEEE.

The input space perturbations are applied to determine the response of the uncertainty measures. In our first experiment, we add additive white Gaussian noise to the input image with various levels of variance (σ). Both model and data uncertainty are characterized for an object detection task. The characterization is performed for the CAMEL dataset. The results are presented in Figure 8.

The results show that the increasing levels of noise decrease the accuracy (mAP) of the tasks. Correspondingly, the uncertainty (class entropy) also increases. A dip follows the σ value of 0.05. This dip occurs as the network fails to produce any positive detections. Thus far, the uncertainty measures we have described are all dependent on actual detections being produced. As the noise level crosses a threshold, the network starts failing. Thus, the uncertainty measure also becomes unreliable in that scenario.

**Figure 9:**
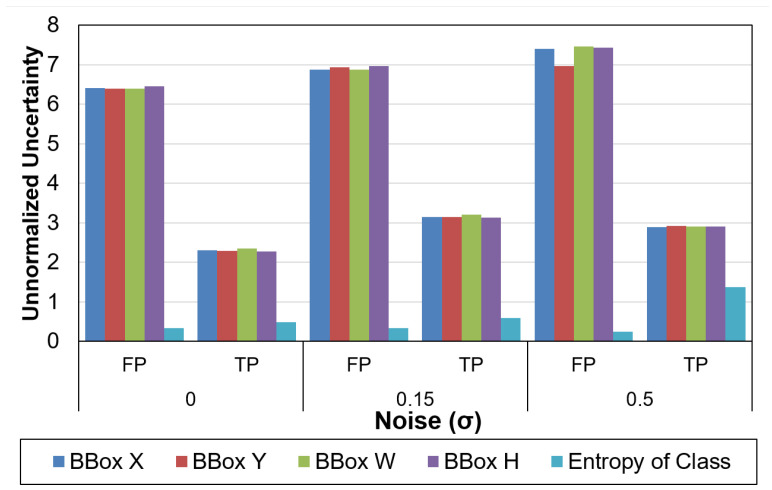
Uncertainty for True Positives (TP) and False Positives (FP) at different levels of sensor noise. Reprinted with permission from ref. [12]. Coprright 2020 IEEE.

At a noise sigma of 0.05, the model/data uncertainty increases by 83%/30%, respectively. The change in model uncertainty is more pronounced than the data uncertainty. The uncertainty for true positives (TP) and false positives (FP) is also examined under various levels of noise as shown in Figure 9. At all levels of noise, the uncertainty values of FP are much higher than TP. For clean data, the uncertainty of TP is 64.1% lower than FP. Thus, in addition to the score of detections, we can use the uncertainty values to remove false positives. This characterization is performed without any feedback.

#### 8.1.2. Multi-Modality

**Figure 10:**
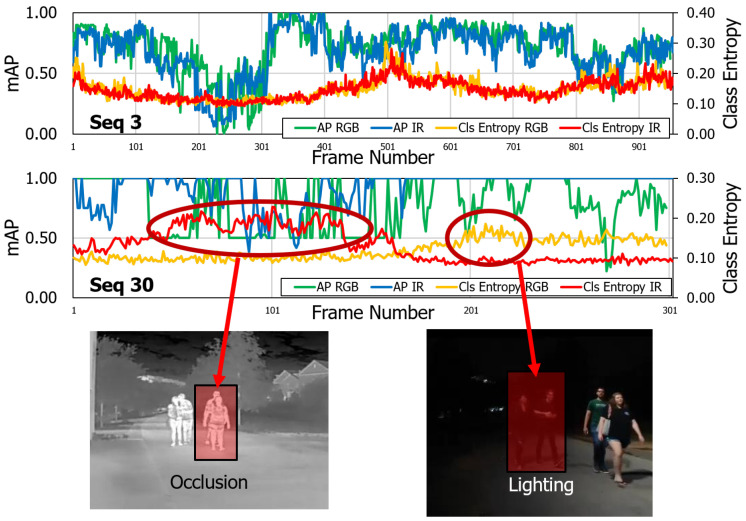
Per-sequence Model Uncertainty Comparison on the CAMEL Test Set. Seq03 is well-lit, so no major fluctuations in uncertainty are observed. In Seq 30, two major spikes are observed in RGB and IR domains due to occlusion and lighting, respectively. Reprinted with permission from ref. [12]. Coprright 2020 IEEE.

We characterize the uncertainty in a multi-modal scenario with RGB and IR modalities available to us. We examine this at a per-sequence level. In Seq03, a well-lit scene, there is no difference in the uncertainty for RGB or IR. In Seq30 (a poorly lit scene), we see a difference in the class entropy. The first observation is that, in the absence of any detections, the class entropy reaches a floor value. This makes uncertainty estimation unsuitable in the case of false negatives. Second, the uncertainty rises due to lighting or occlusion conditions as shown in Figure 10.

#### 8.1.3. Characterization with Feedback

**Figure 11:**
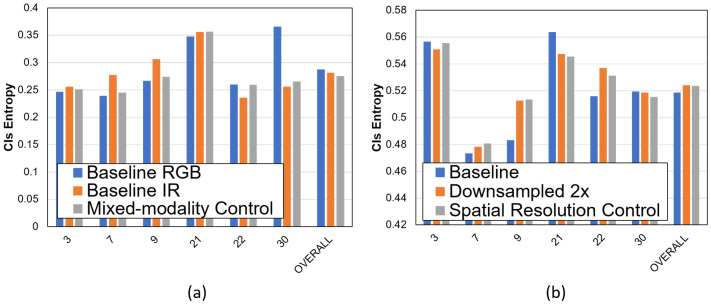
Uncertainty results on CAMEL Dataset. (**a**) With RGB only, IR only and Mixed-modality control (**b**) with RGB, 2× downsampled and Spatial Resolution Control. Reprinted with permission from ref. [12]. Coprright 2020 IEEE.

We characterize the model uncertainty in the confidence-driven feedback imager. We characterize the uncertainty for spatiotemporal resolution control and multi-modality control as shown in Figure 11. In the mixed-modality case, the baselines are RGB only and IR only. For well-lit sequences, there is no difference in the entropy. For poorly lit sequences (Seq 30), the entropy is lower for IR only and mixed modality. The mixed-modality control shows the lowest entropy compared to both baselines.

**Figure 12:**
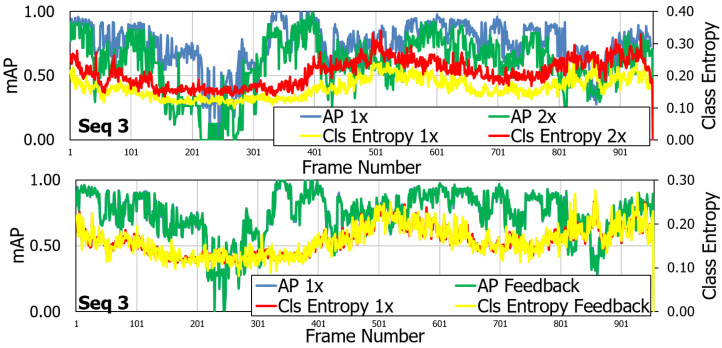
Per-sequence model uncertainty comparison on the CAMEL Test Set. As shown, the task uncertainty for the spatial resolution control is similar to the task uncertainty of the full quality system. Reprinted with permission from ref. [12]. Coprright 2020 IEEE.

The same behavior is observed for spatial resolution control. The entropy for the control is compared to a downsampled by 2× image. The entropy of the feedback control is similar to the baseline RGB system. The advantage is that the feedback control consumes lower bandwidth by 3×. For spatial resolution control, the feedback system shows lower uncertainty than 2× lower resolution system (Figure 12). The feedback system reduces the uncertainty of the detections while transmitting the video at a lower bandwidth.

### 8.2. Uncertainty-Driven Feedback Control

Evaluation of the uncertainty-driven control is performed on the CAMEL dataset and compared with the RGB baseline, RGB-IR Mixed Modality approach [3], and the uncertainty-FP approach described in [12]. In this work, we present the uncertainty-FN and hybrid approach that combines both uncertain-FP and uncertain-FN. In all cases, $th_{conf}$ is set to 0.3 for feedback. The value of $th_{low}$ is set to 0.9 and the value of $th_{high}$ is set to 0.7.

**Figure 13:**
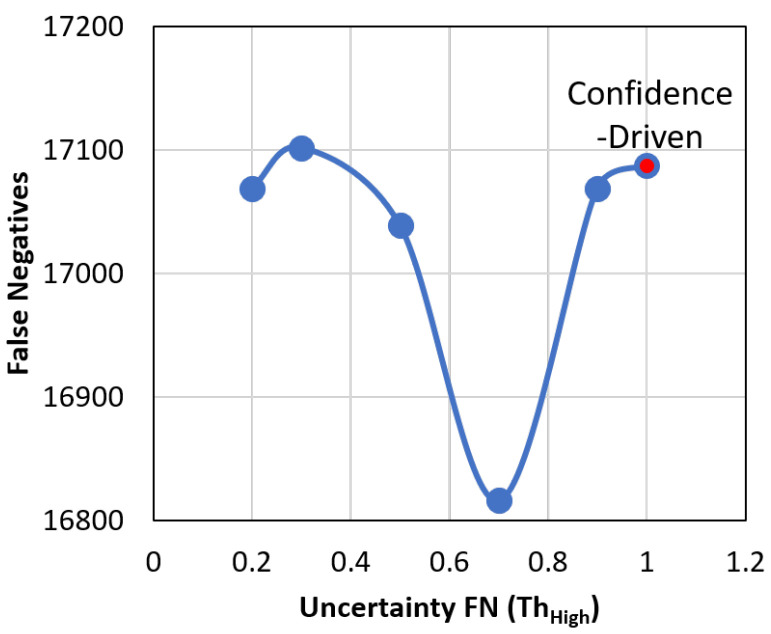
Number of False Negatives on the CAMEL dataset by sweeping the threshold for False Negatives $th_{high}$. A value of 1.0 turns the system to purely confidence-driven.

The threshold for false negative feedback $th_{high}$ is also swept as shown in Figure 13. As it is increased, only the detections with high uncertainty (in addition to the detections with a high score) are considered for feedback. At a value of 1.0, the system becomes purely confidence-driven as no high uncertainty detections are used for feedback. The threshold for confidence $th_{conf}$ is fixed at 0.3.

The object detection and object tracking metrics (AP and MOTA, respectively) show improvement with the addition of uncertainty as a decision criterion as shown in Table 1. The false positives reduce from 587 to 567. The uncertain-FN by itself does not improve FN compared to RGB-IR, but it does improve FN from RGB baseline from 17,307 to 17,266. The hybrid approach shows the best improvement by reducing the FN to 16,816. The mAP and MOTA also improve by 1.1% and 1.1%, respectively.

Similar to the task of object detection and tracking, we also evaluate the uncertainty-driven control on the task of action detection (Table 2) on the UCFSports Dataset. The accuracy metrics improve for the hybrid approach by 1.4%, and the False Negatives reduce by 18 from 74 to 56. Again, the uncertainty-FN by itself does not work well unless it is coupled with Uncertainty-FP. $N_{spatial}$ is set to 2, and $N_{temporal}$ is set to 3 in-line with [4].

**Figure 14:**
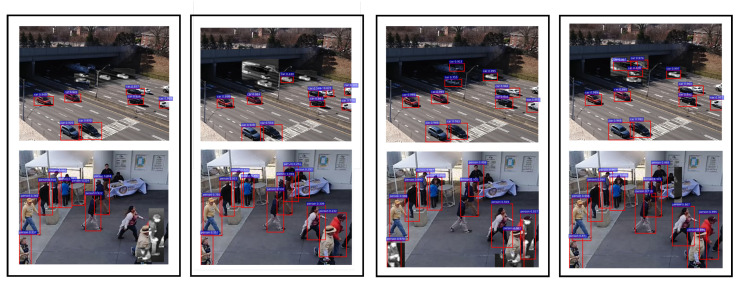
Comparison of confidence-driven and uncertainty-driven control on CAMEL Dataset. Top Row: Seq 09. Bottom Row: Seq 03. Columns from left to right: confidence-driven (output at sensor), uncertainty-driven (output at sensor), confidence-driven (output at end user), and uncertainty-driven (output at end user).

Visually, the confidence-driven and uncertainty-driven feedback controls are shown for the CAMEL dataset in Figure 14. For the car sequence, the uncertainty-driven control manages to detect the cars going in the dark region of the image. Although they have a lower score, they are flagged by their high uncertainty causing the sensor to stay in IR mode. Similarly in the pedestrian sequence, the low score causes the detector to keep switching between RGB and IR in the bottom right section. The uncertainty-driven control is able to detect it in the RGB frame due to its high uncertainty. At the host side, we see the positive effects as the normal network is able to do better detection even though the uncertainty estimation and control happen only at the sensor side.

### 8.3. Separation of Spatial and Temporal Uncertainty

The spatial and temporal uncertainty are compared for two datasets. The UCF Sports dataset contains 10 actions. The videos are trimmed to the action. The actors are fairly centered in the video, and there is little camera motion or motion-induced blurring in the videos. The MOVE dataset, on the other hand, contains a high degree of camera motions and off-center actors. This makes the MOVE dataset a challenging benchmark for video action detection.

**Figure 15:**
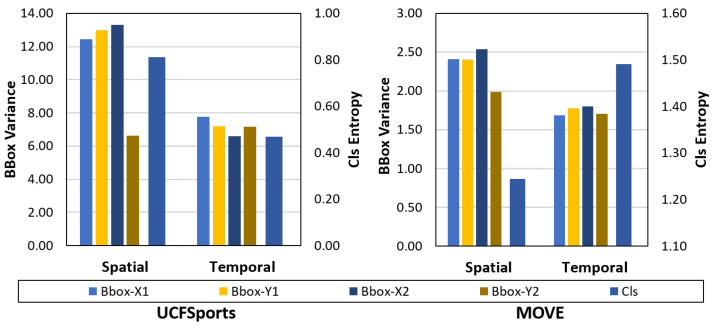
**Left:** Spatial and Temporal Uncertainty for class and location variables on the UCFSports dataset; **Right:** On the MOVE dataset.

The comparison (Figure 15) shows that the spatial uncertainty for the bounding boxes is quite similar. The absolute values differ between datasets due to the different image sizes and actor’s pixel extent. The temporal uncertainty shows an interesting behavior. In terms of absolute value, the temporal uncertainty is higher for the MOVE dataset compared to the UCFSports dataset. Within datasets, the temporal uncertainty is higher vs. spatial uncertainty for the MOVE dataset (1.50 vs. 0.80) while it is lower for the UCFSports dataset (0.45 vs. 0.80). This goes with empirical observations that the localization task is harder in UCFSports while the classification task is harder in the MOVE dataset. Similarly, we observe that the spatial uncertainty is higher for the bounding box variables in both datasets, showing a degree of correlation between bounding box uncertainty and spatial uncertainty.

**Figure 16:**
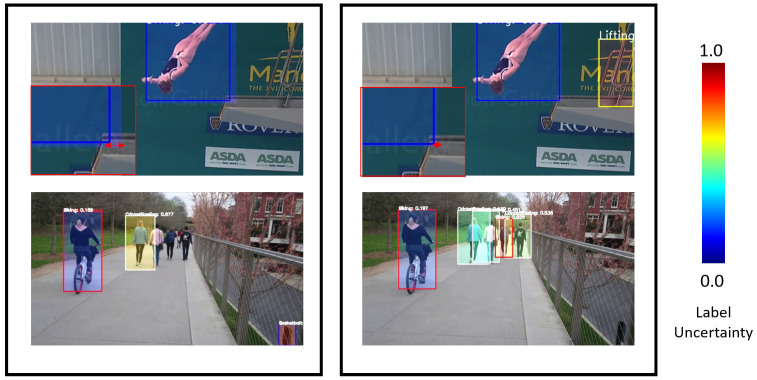
**Left:** Detections with only uncertainty due to spatial features. **Right:** Detections with only uncertainty due to temporal features. The filled extent of the boxes represent the 2σ bbox uncertainty from the mean of the box. The shade of the boxes represents the label uncertainty. The top row is Seq 001 from UCFSports, while the bottom row is Biking01 from MOVE.

The separated spatial and temporal uncertainty is visualized in Figure 16. The spatial uncertainty contributes more to the bounding box uncertainty, while the temporal uncertainty is more pronounced when discussing the class of the detection. We believe that the class is highly dependent on the temporal features for the task of action detection; hence the label uncertainty is also more sensitive to the temporal uncertainty.

#### 8.3.1. Comparing Temporal Aggregators

**Figure 17:**
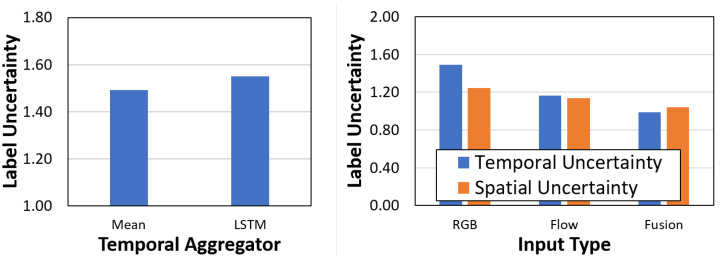
**Left:** Mean vs. LSTM aggregator. **Right:** Comparison with different input types. Evaluated on the MOVE dataset.

The effect of different temporal aggregators is measured. A simple mean aggregator and an LSTM aggregator are used (Figure 17). In the second study, the input modality is changed to study the effect of uncertainty. Between the aggregators, there is little difference in the label uncertainty (both spatial and temporal). The RGB is the baseline configuration with just RGB inputs. In the Flow config, inputs are Brox-Flow images. In the fusion configuration, we have two separate networks for processing RGB and Flow images. The decision of both networks is fused by averaging the scores of the corresponding anchors. The Flow modality shows a lower uncertainty than the RGB one, even though it has a lower AP. The reason is that the Flow modality produces less false positives but is prone to more false negatives, which reduces the AP score. The fusion shows the best AP and lowest uncertainty.

#### 8.3.2. Connecting to the Feedback Control

The separated uncertainty measures show sensitivity to different factors within the video and give us distinct information. Next, we construct a feedback system that utilizes these separated uncertainty measures to drive the control of the sensor. We take a baseline feedback system with action detection task driving spatio-temporal control and construct four configurations to evaluate the uncertainty-driven control.

**Figure 18:**
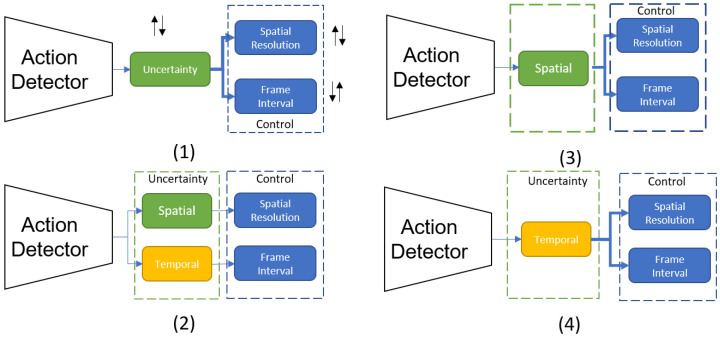
The spatial and temporal uncertainty are connected to the control parameters in 4 different configurations.

In the first, the total uncertainty of the network (calculated by adding dropout layers after every parameter layer) is used to drive both the spatial and temporal control. In configurations (3) and (4), the spatial and temporal uncertainty are connected to both controls, respectively. In configuration (2), the spatial uncertainty is connected to the spatial resolution control while the temporal uncertainty is connected to the temporal resolution control (Figure 18).

The quantitative evaluation (Table 3) shows that, even if the temporal uncertainty is connected to both control knobs, the same accuracy metrics as the baseline are achieved with a fraction of the original cost of multiple MC trials.

## 9. Discussion

The uncertainty of a high-level task DNN was characterized in an active sensor feedback system. It is shown that the uncertainty is minimized in the feedback configurations. A hybrid system that uses uncertainty as a decision criterion was demonstrated. For future work, the aim is to address the limitations of compute complexity. The compute complexity required to obtain the uncertainty estimates is prohibitively high. For each image, we are performing 40 Monte Carlo trials. This number can also change depending on the task we are running. If it is too small, the predictive distribution will not match the true distribution. If it it too large, it will not be feasible to run on a deployed system. Nevertheless, this is an interesting and active area of research. Some lines of research are using distillation to have a small network mimic the uncertainty [37], one-pass uncertainty estimation [38,39], while some advocate developing a trust score calculated using a non-parametric model such as nearest neighbors [40]. It will be interesting to see what developments are presented in that domain.

Additionally, determining the causality of uncertainty is also an interesting research problem that this paper tackles to some extent by separating the uncertainty due to spatial and temporal factors. The thresholds for determining the ROIs based on score and uncertainty are also computed empirically. The threshold may not respond well to changing input distributions; thus, it should be tunable based on an error signal. For example, frequent switching of ROIs may be an indicator of error in the feedback loop, and the threshold can be adjusted accordingly.

## 10. Conclusions

In this work, we have shown that feedback systems that rely solely on the output of the task can lead to deteriorating operations. For detection tasks such as object detection, the error arises due to false positives and false negatives. We show in this work that using uncertainty estimation techniques to measure the reliability of the task output is useful to determine these types of errors. Additionally, we aid the feedback process by using the uncertainty to filter out uncertain detections. The hybrid control scheme involving both uncertainty and score improves the task accuracy. Additionally, for temporal tasks such as action detection, we separate the uncertainty that arises due to spatial/temporal factors. We show that, in a practical system, just using the temporal uncertainty is enough to guarantee reliable feedback operation.

## Figures and Tables

**Table 1:** Accuracy metrics on the CAMEL dataset.

	Detection (mAP) ↑	Tracking (MOTA) ↑	Bandwidth (Mbps) ↓	TP ↑	FP ↓	FN ↓
RGB	0.223	0.221	61.9	5357	609	17,307
RGB-IR [3]	0.233	0.235	52.8	5577	587	17,087
Uncertain-FP [12]	0.234	0.235	53.2	5608	567	17,056
**This Work**						
Uncertain-FN	0.223	0.230	53.3	5389	589	17,266
Hybrid	0.244	0.246	53.2	5848	571	16,816

**Table 2:** Accuracy metrics on the UCFSports dataset.

	Frame mAP ↑	Bandwidth (Mbps) ↓	TP ↑	FP ↓	FN ↓
No Feedback	76.9	210	3132	68,654	74
Spatio-Temporal Control					
Confidence-Driven	77.3	29.03	3132	66,315	74
Uncertainty-FP	78.3	28.77	3129	65,128	77
Uncertainty-FN	78.4	29.10	3077	66,314	129
Hybrid	78.7	29.12	3150	64,318	56

**Table 3:** Ablation Study on Feedback Configurations with Spatial and Temporal Uncertainty.

	Frame mAP ↑	Bandwidth (Mbps) ↓	Compute (GFLOPS) ↓
Config 1	78.7	29.12	17.4 × T
Config 2	78.6	28.76	17.4 × T
Config 3	78.5	28.55	17.4 × T
Config 4	78.1	29.97	14.9 + 2.52 × T

T is the number of Monte Carlo Trials.

## Data Availability

Datasets used in this study are openly available at https://camel.ece.gatech.edu, https://www.crcv.ucf.edu/data/UCF101.php (both accessed on 5 April 2021).
